# Chronic Exposure of Gingival Fibroblasts to TLR2 or TLR4 Agonist Inhibits Osteoclastogenesis but Does Not Affect Osteogenesis

**DOI:** 10.3389/fimmu.2020.01693

**Published:** 2020-07-23

**Authors:** Gerasimos D. Karlis, Emily Schöningh, Ineke D. C. Jansen, Ton Schoenmaker, Jolanda M. A. Hogervorst, Henk A. van Veen, Carolyn G. J. Moonen, Katarzyna B. Łagosz-Ćwik, Tim Forouzanfar, Teun J. de Vries

**Affiliations:** ^1^Department of Periodontology, Academic Centre for Dentistry Amsterdam (ACTA), University of Amsterdam and Vrije Universiteit, Amsterdam, Netherlands; ^2^Amsterdam University College, Amsterdam, Netherlands; ^3^Department of Oral Cell Biology, Academic Centre for Dentistry Amsterdam (ACTA), University of Amsterdam and Vrije Universiteit, Amsterdam, Netherlands; ^4^Department of Cell Biology and Histology, Electron Microscopy Centre Amsterdam, Academic Medical Center, Amsterdam UMC, Amsterdam, Netherlands; ^5^Department of Biochemistry, Microbiology and Immunology, University of Ottawa, Ottawa, ON, Canada; ^6^Department of Microbiology, Faculty of Biochemistry, Biophysics and Biotechnology, Jagiellonian University, Kraków, Poland; ^7^Department of Oral and Maxillofacial Surgery and Oral Pathology, Amsterdam UMC, Amsterdam, Netherlands

**Keywords:** chronic inflammation, toll-like receptors, periodontitis, TNF-α, bone resorption, osteoclasts, osteoblasts, innate immunity

## Abstract

Chronic exposure to periodontopathogenic bacteria such as *Porphyromonas gingivalis* and the products of these bacteria that interact with the cells of the tooth surrounding tissues can ultimately result in periodontitis. This is a disease that is characterized by inflammation-related alveolar bone degradation by the bone-resorbing cells, the osteoclasts. Interactions of bacterial products with Toll-like receptors (TLRs), in particular TLR2 and TLR4, play a significant role in this chronic inflammatory reaction, which possibly affects osteoclastic activity and osteogenic capacity. Little is known about how chronic exposure to specific TLR activators affects these two antagonistic activities. Here, we studied the effect of TLR activation on gingival fibroblasts (GF), cells that are anatomically close to infiltrating bacterial products in the mouth. These were co-cultured with naive osteoclast precursor cells (i.e., monocytes), as part of the peripheral blood mononuclear cells (PBMCs). Activation of GF co-cultures (GF + PBMCs) with TLR2 or TLR4 agonists resulted in a weak reduction of the osteoclastogenic potential of these cultures, predominantly due to TLR2. Interestingly, chronic exposure, especially to TLR2 agonist, resulted in increased release of TNF-α at early time points. This effect, was reversed at later time points, thus suggesting an adaptation to chronic exposure. Monocyte cultures primed with M-CSF + RANKL, led to the formation of bone-resorbing osteoclasts, irrespective of being activated with TLR agonists. Late activation of these co-cultures with TLR2 and with TLR4 agonists led to a slight decrease in bone resorption. Activation of GF with TLR2 and TLR4 agonists did not affect the osteogenic capacity of the GF cells. In conclusion, chronic exposure leads to diverse reactions; inhibitory with naive osteoclast precursors, not effecting already formed (pre-)osteoclasts. We suggest that early encounter of naive monocytes with TLR agonists may result in differentiation toward the macrophage lineage, desirable for clearing bacterial products. Once (pre-)osteoclasts are formed, these cells may be relatively insensitive for direct TLR stimulation. Possibly, TLR activation of periodontal cells indirectly stimulates osteoclasts, by secreting osteoclastogenesis stimulating inflammatory cytokines.

## Introduction

Periodontitis is a plaque-related inflammatory disease of the tooth-supporting tissues, leading to alveolar bone resorption which, eventually, can lead to tooth loss (1, 2). It is initiated by a disturbed balance between the host immune response and the bacterial load, modified by several factors such as lifestyle, genetics, and individual variation in the subgingival microbiome (3–5). Initially, the inflammatory response plays a protective role, orchestrated to eliminate the damaging stimulus and restore symbiosis (6). However, in patients with periodontitis, this inflammatory reaction is often chronic, leading to the irreversible alveolar bone resorption, which is mediated by bone-resorbing cells; the multinucleated osteoclasts (7).

The first line of host defense to micro-organisms or their products is initiated by the innate immune response. It is conceivable that gingival fibroblasts (GF), the predominant cell type of the alveolar bone-lining mucosa (gingiva), interact constantly with molecules from the oral microflora. These fibroblasts express receptors that sense the presence of microbes and substances released by these microbes. These receptors are referred to as “Pattern recognition receptors” (PRRs) since they recognize molecular patterns that are commonly present on many micro-organisms. One of the functions of the innate immune response is the recognition of pathogen-associated molecular patterns (PAMPs) by PRRs, including the Toll-like receptors (TLRs) (8). Up till now, ten TLRs (TLR 1-10) have been identified in humans which respond to these PAMPs (9, 10). Each TLR responds to specific PAMPs, however mainly a combination of them is required to be activated.

All of these TLRs are expressed in periodontal tissues (11, 12). TLR2 and TLR4 are the most extensively researched receptors of the TLR family in relation to periodontitis in mice and men (10, 13–16). This derives from the fact that TLR4 is stimulated by lipopolysaccharides (LPS) (17), the major glycolipid membrane component of the Gram-negative bacteria, such as the keystone periodontopathogenic bacterium *Porphyromonas gingivalis* (18, 19). TLR2 is involved in the recognition of cell-wall components of Gram-negative and Gram-positive bacteria. The participation of these two specific members of the TLR family in the triggering of the innate immune response in periodontitis patients is already established (10–12, 20, 21). Accordingly, higher expression of these receptors has been found in the periodontal tissues of periodontitis patients, in comparison with healthy controls (12, 22).

TLR2 and TLR4 are expressed in the periodontal tissues, and among them on GF (23, 24). GF play an important role in processes associated with bone remodeling such as the induction and inhibition of osteoclast formation (25, 26). Osteoclasts, the cells that are responsible for bone resorption, are derived from the monocyte lineage and express TLRs which respond to PAMPs (27). It has been shown that ligature and injection-induced periodontitis in mice is regulated through the activation of the TLR4 and TLR2 receptors (28, 29). However, there is also evidence that shows that the *in vitro* activation of human osteoclast precursors with TLR agonists results in the inhibition of osteoclastogenesis (30). Besides inhibition of osteoclasts, chronic TLR2 activation plays a significant role in T cell proliferation, mediated by GF or monocytes, resulting in the production of proinflammatory cytokines by human monocytes (24).

GF can also be stimulated into the osteogenic lineage (31). Little is known about the effect of TLR activation of these cells in the context of osteogenesis. It has been shown that TLR2 agonist (Pam3CSK4 or mutant *E. coli*) slightly enhances osteogenesis in human primary osteoblasts (32). However, the dose of the agonists was low (1 μg/mL and 1 ng/mL, respectively) and it was not clearly stated if the agonists were added only once or with every refreshment of the media. Others found that TLR4 has an inhibitory effect on osteogenesis in murine bone marrow mesenchymal stem cells (33). Recently, it was shown that *in vitro* TLR4 activation in high doses (10 μg/mL) inhibits the osteogenic potential of human periodontal ligament cells (34).

Although periodontitis is a chronic inflammation, and the expression of TLR2 and TLR4 is aberrant in the GF (24, 35), the effect of the activation of these specific TLRs on osteoclastogenesis and osteogenesis is only evaluated after short (<60 h) stimulation (36–40), and scarcely on cells derived from human periodontal tissues (34, 41–43).

To the best of our knowledge, this is the first study that evaluated the effect of chronic exposure of specific TLR2 and TLR4 agonists, molecules that activate TLR2 and TLR4, both on osteogenesis, in presence of human GF, and on osteoclastogenesis, in GF stimulated peripheral blood mononuclear cell (PBMC) cultures. Since TLR stimulators may also affect precursors of osteoclasts or multinucleated osteoclasts, we studied these effects on monocytes that were cultured with macrophage stimulating factor (M-CSF) and receptor activator of nuclear factor kappa-B ligand (RANKL) for 1 week (pre-osteoclasts) and for 2 weeks (osteoclasts) followed by 2 vs. 1 week of TLR agonist exposure, to assess the effect on osteoclast differentiation and activity on bone slices. We hypothesized that the triggering of these TLRs would result in an induction of osteoclastogenesis and an inhibition of osteogenesis.

## Materials and Methods

### Gingival Fibroblasts

GF were obtained from 6 systemically healthy individuals (age 22–38 years) who underwent extraction of a third molar (wisdom tooth). No overt signs of gingival inflammation and periodontitis were present (pockets ≤ 3 mm without bleeding). Sampling from the donors was conducted at VU University Hospital (Vrije Universiteit, Amsterdam, The Netherlands). All the individuals signed informed consent and samples were coded to guarantee the anonymity of the donors as required by Dutch law. Researchers handling the fibroblasts (G.D. Karlis and T.J. de Vries) could not retrieve the identity of the donors.

With the use of a scalpel-knife, free gingiva and part of the interdental gingiva were cut off the tooth. The tissue fragments were washed twice in culture medium (Dulbecco's minimal essential medium (DMEM, Gibco BRL, Paisley, Scotland) supplemented with 10% fetal calf serum (FCS, HyClone, Logan, USA), and 1% antibiotics (100 U/mL penicillin, 100 mg/mL streptomycin, and 250 ng/mL amphotericin B [Antibiotic antimycotic solution, Sigma, St. Louis, MO, USA]) and cultured in a humified atmosphere of 5% CO_2_ in air at 37°C. For the current study, GF of passages 4–6 were used.

### Blood Cell Isolation

Buffy coats (Sanquin, Amsterdam, The Netherlands) of healthy donors were diluted 1:1 in 1% PBS-citrate (pH 7.4). Thereafter, 25 mm of diluted blood was carefully layered on 25 mL Lymphoprep (Axisshield Po CAS, Oslo, Norway) and centrifuged for 30 min at 800 x G without brake. The interphase containing the PBMCs was collected and washed three times in 1% PBS-citrate and finally recovered in culture medium.

### Monocyte Isolation

CD14+ monocytes retrieved from peripheral blood were used in experiments where osteoclasts were grown using M-CSF and RANKL instead of fibroblasts and PBMCs. Here, CD14+ cells were isolated using CD14+ microbeads (Miltenyi, Bergisch Gladbach, Germany) according to a previously described method (44).

### TLR Agonist Titrations

Optimal cell densities (ratio) of GF and PBMC were previously established by our group (25). GF (1.5 x 10^4^ per well, *n* = 3) were seeded in duplicate and allowed to attach overnight in 48-well plates. 5 x 10^5^ PBMCs were seeded in duplicate in co-culture with GF. To assess the optimal concentration of TLR agonists, co-cultures for osteoclastogenesis cultures as described above or GF monocultures for osteogenesis (3.0 x 10^4^ cells per well) (31) were cultured and maintained in a humidified atmosphere of 5% CO_2_ in ambient air at 37°C. Cultures were refreshed every 3–4 days. A titrated concentration of TLR2 ligand (10 ng/mL, PAM2CSK4, #14E14-MM, Invivogen, San Diego, CA, USA), TLR4 ligand (10 ng/mL, LPS- *Porphyromonas gingivalis*, Ultrapure, Version #14F18-MM, Invivogen), or a combination of both, was added to the culture media at the start of the experiment (*n* = 6) and with every subsequent culture media refreshment (every 3–4 days). For assessing the effect of TLR2 and TLR4 activation on TLR activation in general, a TLR2 and TLR4 targeting LPS from *Porphyromonas gingivalis* was used (Catalog number #tlrl-pglps, Invivogen). This LPS activates TLR2 at 10 ng ng/mL and TLR4 from 100 ng/mL. Both these concentrations were used in the relative experiments.

Control conditions contained culture media without TLR agonists but included similar additions of a vehicle (sterile water). PBMCs were also seeded in high-density cultures at 1 x 10^6^ PBMCs per 96-well plate (*n* = 4).

CD14+ monocytes were cultured for 3 days in M-CSF (25 ng/mL), followed by 10 ng/mL M-CSF, and 10 ng/mL RANKL until 21 days. Pre-osteoclasts at 7 days, or early osteoclasts at 14 days, received TLR agonists for the remaining 14 or 7 days respectively.

### TRAcP Staining

After 21 days, cells were fixed in 4% PBS-buffered formaldehyde for 10 min and washed with PBS. Cells were stained with a TRAcP staining (Sigma-Aldrich) according to the manufacturer's protocol. Nuclei were counterstained with 4′,6-diamidino-2-phenylindole (DAPI) for 5 min. A combination of light and fluorescence microscopy (Leica DFC320; Leica Microsystems, Wetzlar, Germany) was used to count the TRAcP + multinucleated cells (MNCs) and cells were considered to be osteoclasts when TRAcP positive with at least three nuclei. Five standardized areas per well were analyzed at a magnification of 20 x to count the number for the number of MNCs containing at least three nuclei and are expressed as MNCs/ well.

### Bone Resorption

Bone resorption was analyzed in cultures on bone after a culture period of 3 weeks. After this period, the cells present on the bovine cortical bone slices were removed with 0.25 M NH_4_OH. The slices were washed in distilled water, incubated in a saturated alum solution, washed in distilled water, and stained with Coomassie Brilliant blue. The surface areas of individual resorption pits were measured using Image-Pro Plus software (Media Cybernetics, Silver Spring, MD).

### Osteogenesis

Osteogenesis assays were performed as previously described (14, 31). Briefly, GF were seeded in 48-wells plates (3 x 10^4^ cells/well). Culture medium (0.4 mL per well) was replaced twice per week. The culture medium contained 50 μg/mL ascorbic acid (Sigma) and 10 nM β-Glycerophosphate (Sigma), which are conductive to mineralization (further referred to as mineralization medium). Water as solvent control, TLR2 agonist, TLR4 agonist, or the combination of these agonists was added for 21 days. Cells were harvested for quantitative PCR analysis by adding RNA lysis buffer (Qiagen, Hilden Germany) containing 1% β-mercaptoethanol and were stored at −80°C until RNA extraction. Cells for alkaline phosphatase activity and DNA measurements were lysed in Milli-Q water and stored at −20°C. The cells for the mineralization assay were fixed with 4% PBS buffered formaldehyde for 10 min and were stored with PBS at 4°C.

In order to evaluate the osteogenic capabilities of the TLR agonists *in vitro*, we measured the calcium deposition (μg/mL), in the 4 different conditions (with TLR2, TLR4, TLR2 + TLR4, and without, respectively) and at 4 different time-points (*t* = 0, 7, 14, 21 days).

### Alkaline Phosphatase

Alkaline phosphatase activity (ALP) was measured in lysates from cells that were cultured with mineralization medium. Cells were harvested at days 0 and 14 of culturing. Cells were washed with PBS and lysed with 200 μL Milli-Q water and were frozen in −20°C for storage. After three freeze-thaw cycles, samples were collected by scraping. ALP was measured according to the method described by Lowry (45), using 4-nitrophenyl phosphate disodium salt (Merck, Darmstadt, Germany) at pH 10.3, as a substrate for ALP. Absorbance was measured at 405 nm with a Synergy HT spectrophotometer (BioTek Instruments Inc., Winooski, VT, USA). DNA was measured in the same lysate using CyQuant Cell Proliferation Assay Kit (Molecular Probes, Leiden, The Netherlands). Fluorescence was measured at 485 nm excitation and 528 nm emission with a Synergy HT spectrophotometric microplate reader. Alkaline phosphatase was expressed as μmol/ng DNA.

### Alizarin Red Staining

Mineral deposition, in triplicate wells per donor, was analyzed after 21 days of culturing. The cells were fixed for 10 min in 4% formaldehyde and rinsed with Milli-Q water before adding 300 μL of 2% Alizarin Red solution at pH 4.3 (Sigma-Aldrich, St. Louis, MO, USA). After incubation of 15 min at room temperature, the cells were washed with Milli-Q water and air-dried. Red nodules were a sign of mineral deposition.

### Calcium Quantification

Samples for calcium deposition assay were collected on days 0, 14, 21. First, 1 mL of 0.5 M acetic acid was added. Secondly, calcium was extracted by shaking the samples overnight. Calcium content was measured in the extraction solution using the ortho-cresolphthalein complexone (OCPC) method (46). Absorbance was measured at 570 nm in a microplate reader (BioTek Synergy HT).

### Scanning Electron Microscopy

Mineralization assays were performed on cells that were grown on glass insert slides, in the presence or absence of TLR agonists and mineralization medium. At 21 days, cells were washed in cacodylate buffer, fixed in MacDowells fixative, and dehydrated in steps of increasing percentage of ethanol. Gold-sputtered preparations were analyzed with a Zeiss Sigma 300 FESEM (Carl Zeiss Microscopy GmbH, Jena, Germany) scanning electron microscope.

### ELISA

Conditioned medium was taken from mono- and co-cultures (*n* = 6) at 3, 7, and 21 days. Enzyme-linked immunosorbent assays (ELISA, R&D Systems, Minneapolis, MN, USA) were used for the detection of human tumor necrosis factor alpha (TNF-α) following the manufacturer's instructions.

### Real-Time Quantitative PCR

After 7 and 21 days of culturing, RNA was extracted from samples using a commercial spin-column kit (RNeasy Mini kit, Qiagen, Düsseldorf, Germany) according to the manufacturer's protocol. RNA concentration was measured with Synergy HT spectrophotometer (BioTek Instruments Inc., Winooski, VT, USA). One hundred nanograms RNA was used in the reverse transcriptase reaction which was performed according to the manufacturer's instructions of the MBI Fermentas cDNA synthesis kit (Vilnius, Lithuania), using both the Oligo(dT)18 and the D(N)6 primers. The Primer Express software, version 2.0 (Applied Biosystems, Foster City, CA, USA) (Table 1) was used to design the Real-time PCR primers.

**Table 1:** Primer sequences used for RT-qPCR experiments.

**Gene**		**Primer sequence**	**Ensembl gene ID**
TRAcP	Forward	5′ CACAATCTGCAGTACCTGCAAGGAT 3′	ENSG00000102575
	Reverse	5′ CCCATAGTGGAAGCGCAGATA 3′	
NFATc1	Forward	5′ CATGCGAGCCATCATCGA 3′	ENSG00000206439
	Reverse	5′ TGGGATGTGAACTCGGAAGAC 3′	
TNF-α	Forward	5′ CCCAGGGACCTCTCTCTAATCA 3′	ENSG00000111956
	Reverse	5′ GCTTGAGGGTTTGCTACAACATG 3′	
RUNX2	Forward	5′ CCAGAAGGCACAGACAGAAGCT 3′	ENSG00000124813
	Reverse	5′ AGGAATGCGCCCTAAATCACT 3′	
ALP	Forward	5′ GCTTCAAACCGAGATACAAGCA 3′	ENSG00000162551
	Reverse	5′ GCTCGAAGAGACCCAATAGGTAGT 3′	
Osteonectin	Forward	5′ GCCCAGCGGTGCAGAGT 3′	ENSG00000196104
	Reverse	5′ GGCTCCCAGCCATTGATACA 3′	
TLR2	Forward	5′ GGCTTCTCTGTCTTGTGACCG 3′	ENSG00000137462
	Reverse	5′ GAGCCCTGAGGGAATGGAG 3′	
TLR4	Forward	5′ CTGCAATGGATCAAGGAACCAG 3′	ENSG00000136869
	Reverse	5′ CCATTCGTTCAACTTCCACCA 3′	
β2-microglobulin	Forward	5′ CGGGCATTCCTGAAGCTGA 3′	ENSG00000106927
	Reverse	3′ GGATGGATGAAACCCAGACACATAG 3′	

Real-time PCR was performed on the ABI PRISM 7000 (Applied Biosystems). The reactions were performed with 5 ng cDNA in a total volume of 25 mL containing SYBR Green PCR Master Mix, consisting of SYBR Green I Dye, AmpliTaq Gold DNA polymerase, dNTPs with dUTP instead of dTTP, passive reference and buffer (Applied Biosystems) and 300 nM of each primer. After an initial activation step of the AmpliTaq Gold DNA polymerase for 10 min at 94°C, 40 cycles were run of a two-step PCR consisting of a denaturation step at 94°C for 30 s and annealing and extension step at 60°C for 1 min. Subsequently, the PCR products were subjected to melting curve analysis to test if any unspecific PCR products were generated. The PCR reactions of the different amplicons had equal efficiencies. β2-microglobulin was used as the housekeeping gene. Expression of this gene was not affected by the experimental conditions. Samples were normalized for the expression of β2-microglobulin by calculating the ΔCt, (Ct_gene of interest_ -Ct,_β2−*microglubulin*_) and expression of the different genes is expressed as the mean relative fold expression 2^−(ΔCt)^.

### Statistics

GraphPad Prism software (version 8.3.0, La Jolla, CA, USA) was used to analyze the data sets. Means and standard deviations (SD) were calculated and used for the presentation of the data in figures. All the data were analyzed with one-way ANOVA followed by Tukey's multiple comparison test. Tests were performed over the 4 (osteoclastogenesis) or 5 (osteogenesis) conditions per time point and per condition over time. Differences were considered significant at *p* < 0.05.

## Results

### Chronic Exposure to TLR Agonists Decreases the Osteoclastogenic Capacity of GF-PBMC Co-cultures

**Figure 1:**
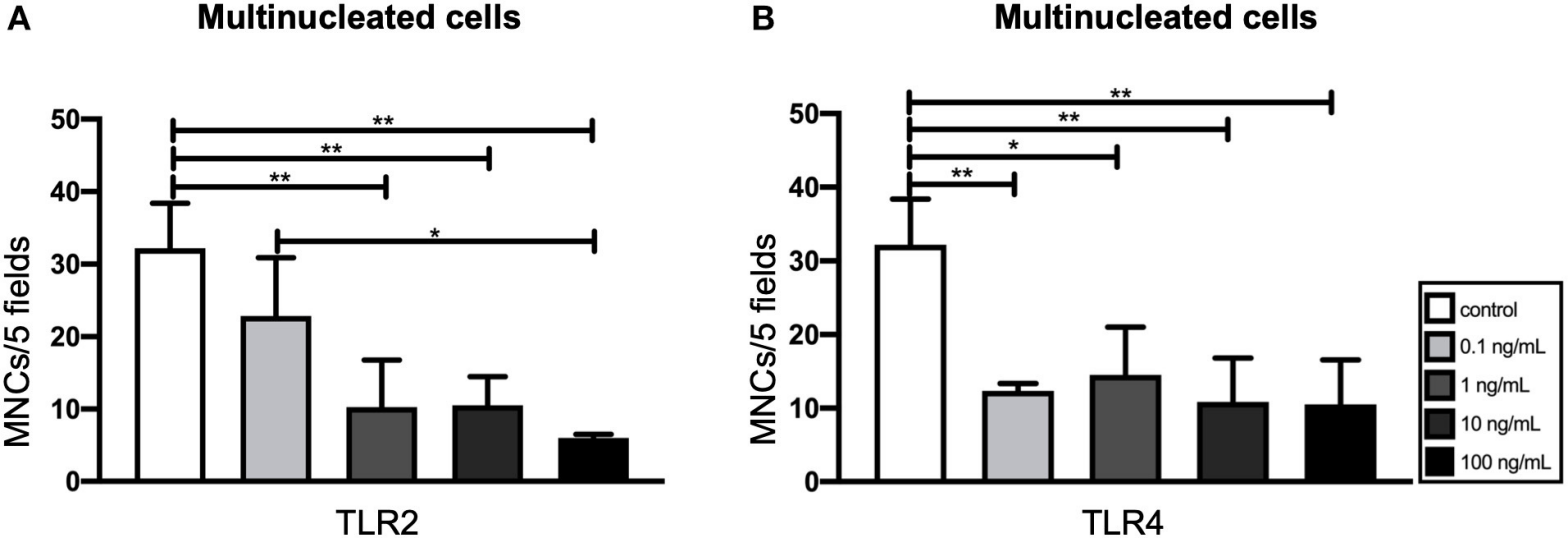
Effect of Toll-like receptor (TLR) agonist concentrations on the osteoclastogenic and the osteogenic capacity of gingival fibroblasts (GF). **(A)** The number of multinucleated cells formed in the presence of TLR2 agonist (0.1–100 ng/ml). **(B)** The number of multinucleated cells formed in the presence of TLR4 agonist (0.1–100 ng/mL). Titration experiments were performed using three different GF donors in duplicates, average results ±SD are shown. Significant results for **(A)** and **(B)** are shown (black bars). *p < 0.05, **p < 0.01.

In order to identify the most suitable concentration of TLR agonists for the experiment, various concentrations of TLR2 and TLR4 were tested (0.1, 1, 10, and 100 ng/mL). For osteoclastogenesis experiments, GF and PBMCs were co-cultured for 21 days with or without TLR agonists. In order to identify osteoclasts, TRAcP+ cells with more than 3 nuclei were counted and categorized into three different groups (3–5, 6–10, and ≥11 nuclei) (47). Because the vast majority of the multinucleated cells had 3–5 nuclei, all three categories were merged into one category. The addition of TLR2 and TLR4 agonists appeared to be associated with the formation of fewer multinucleated cells (Figures 1A,B). This effect was statistically significant for all concentrations, except for the 0.1 ng/mL TLR2 condition (Figure 1A).

### TLR2 Agonist Decreases the Number of Multinucleated Cells in GF-PBMC Co-cultures

**Figure 2:**
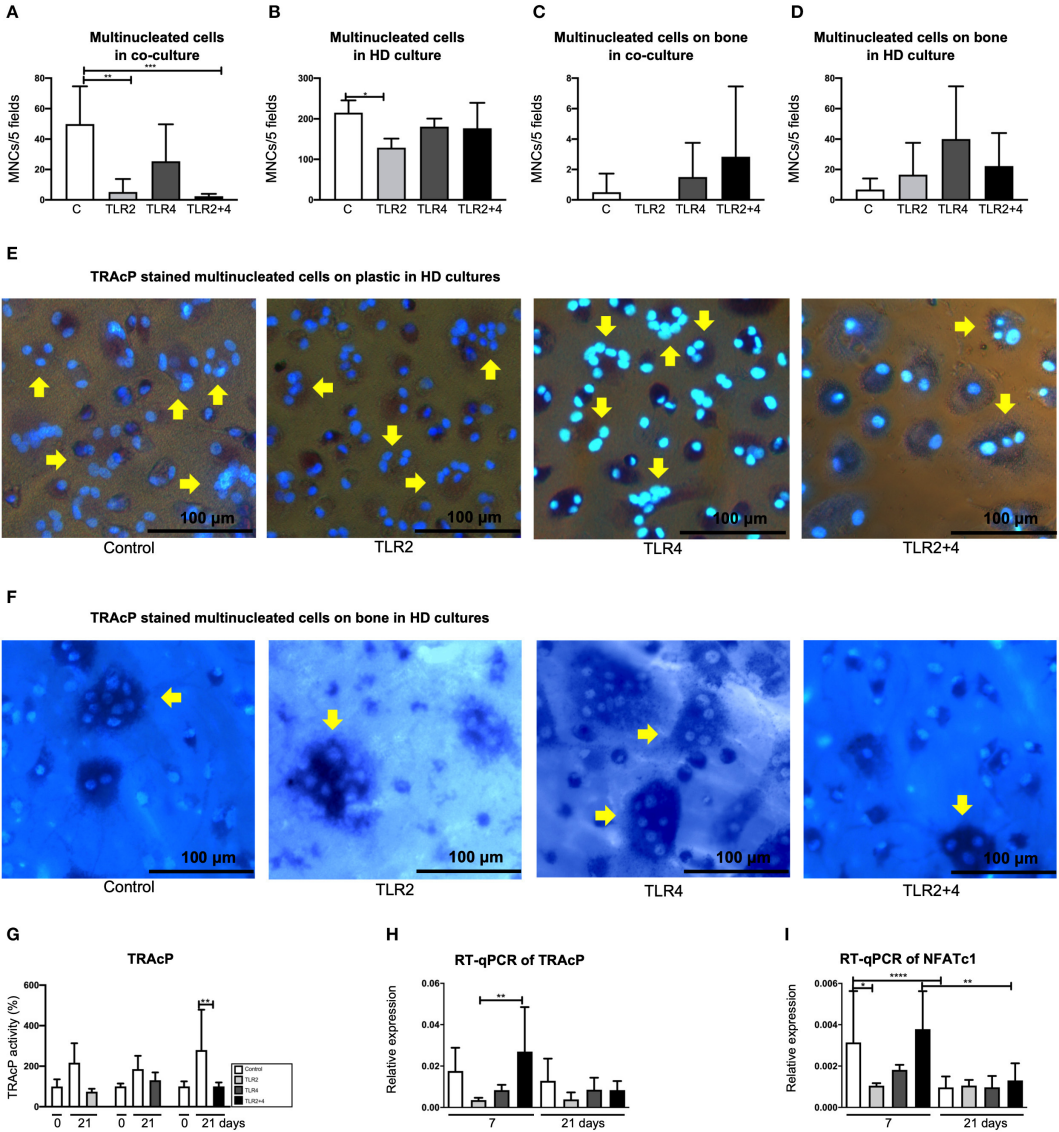
TLR2 agonist decreases the number of multinucleated cells in GF-PBMC co-cultures and PBMC high-density cultures. The number of multinucleated cells is presented in **(A–D)**. The concertation of the agonists was 10 ng/mL. Osteoclast formation is particularly decreased in the presence of TLR2 (light gray bars, **A,B**) and TLR2+4 agonists (black bars, **A**). **(C)** Multinucleated cells were counted on bone slices but they were in very low numbers. **(D)** In high density cultures, multinucleated cells were a more common finding but without any difference between the conditions. Cells were stained for tartrate-resistant acid phosphatase (TRAcP, purple) and counterstained with DAPI (blue), in order to detect the multinucleated cells (depicted with yellow arrows); on plastic **(E)** and on bone **(F)**. All micrographs are representatives for three independent experiments with six different GF sources. Scale bar represents 100 μm. **(G)** TRAcP enzyme activity was measured by three different conditions, each with separate 0 days and a 21 days control samples. TRAcP enzyme activity is significantly decreased at day 21 at the TLR2+4 condition in comparison with the control. **(H,I)** Real-time quantitative PCR was performed for the genes of TRAcP and NFATc1). *n* = 6 GF cultures per condition in duplicates, average results ±SD are shown. For the HD cultures *n* = 4. Significant results for **(A–D)** and **(G–I)** are shown (black bars). *p < 0.05, **p < 0.01, ***p < 0.005, ****p < 0.001. ^a^High density culture.

Based on the results of the titration experiment (Figure 1), a concentration of 10 ng/mL of TLR agonists was chosen for further experiments. To analyze whether activation of both TLR2 and TLR4 would lead to increased sensitivity, a condition of 10 ng/mL of both TLR2 and TLR4 agonist was included in all further experiments. GF were co-cultured with PBMCs for 21 days. The cells were stained for TRAcP activity and cells with 3 or more nuclei were counted as multinucleated cells, in 5 standardized fields per well. The presence of TLR2 and TLR2 + 4 agonists was significantly associated with fewer multinucleated cells, in comparison with the control (Figure 2A), suggesting that TLR2 agonist decreased the number of MNCs in these co-cultures. Apart from GF-PBMC co-cultures, there is another way to culture multinucleated cells in the absence of cytokines. When PBMCs are plated at a high density, multinucleated cells will form (48). Here, T-cells seem important for providing the signals for the formation of multinucleated cells (49). Also, when applying this method, less multinucleated cells formed when TLR2 agonist was added (Figure 2B). Multinucleated cells were counted on bovine bone slices. The presence of these cells on the bone was rare (Figure 2C). Multinucleated cells on bone found to be more often present in high-density cultures compared to normal density co-cultures on bone. No significant differences were found (Figure 2D) on bone slices, suggesting that the surface may modulate TLR agonist's activity. Representative micrographs of TRAcP+ cells on plastic and on bone are shown in Figures 2E,F, respectively. The TRAcP enzyme was also quantified at baseline (day 0) and day 21 (Figure 2G). TRAcP enzyme was statistically decreased at day 21 under the effect of TLR2+4 in comparison with the control. The expression of TRAcP mRNA was measured also with Real-Time qualitative PCR (Figure 2H). A trend of less expression in the conditions of TLR2 and TLR4 in comparison with the control was found without being statistically significant. The only difference was found between TLR2 and TLR2+4 at day 7, with the TLR2+4 being elevated compared to TLR2 (Figure 2H). The gene expression of NFATc1, a crucial transcription factor for osteoclast formation (50) was measured (Figure 2I). Expression of NFATc1 was significantly lower for TLR2 in comparison with the control, at day 7. At day 21, the expression of the gene was significantly lower in the conditions of control and TLR2 + 4 in comparison with day 7.

### Secretion of TNF-α: Early TLR2 Agonist Responses and Nullification Over Time

**Figure 3:**
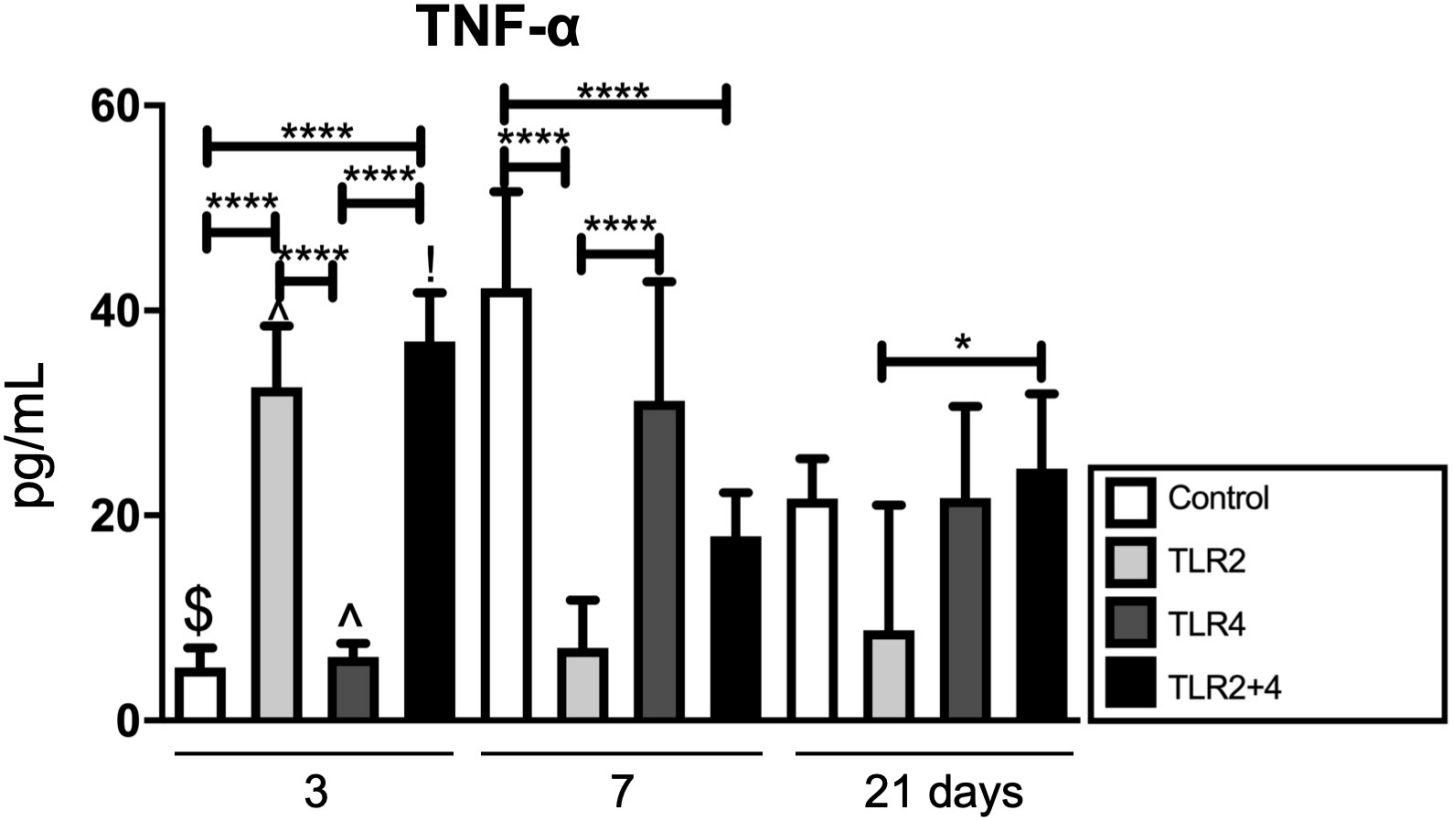
TLR2 agonist has an evident effect on TNF-α; increasing in early timepoints, reversed later. TNF-α is significantly elevated at day 3 in all conditions containing TLR2 agonist (light gray and black); at day 7, these conditions are significantly decreased in comparison with the control. At day 21, the are no differences anymore between TLR conditions and control. *n* = 6 GF cultures per condition in duplicates, average results ±SD are shown. Significant results are shown (black bars). *p < 0.05, ****p < 0.001, ^*$*^*p* < 0.01 for the same condition between day 3 and day 7, day 3 and day 21 as well as day 7 and day 21, ^∧^*p* < 0.001 for the same condition between day 3 and day 7, as well as day 3 and day 21, !*p* < 0.001 for the same condition between day 3 and day 7.

We previously described, that TLR activation results in the production of inflammatory cytokines (24). We next measured TNF-α in the supernatant of GF-PBMC co-cultures (Figure 3). Since little is known about the production of TNF-α in chronically TLR agonist exposed cultures, we measured TNF-α at 3 different time points; at 3, 7, and 21 days. At day 3, the levels of TNF-α were significantly elevated in the groups that contained TLR2 agonists (TLR2 and TLR2+4) in comparison with the control (Figure 3). At day 7, these results were reversed, where the levels of TNF-α of the control and TLR4 conditions were significantly higher than when TLR2 agonist was added. On day 21, secretion of TNF-α was significantly lower when TLR2 agonist was added compared to TLR2+4 agonists.

### Effect of TLR Agonists on the Number of Multinucleated Cells and Bone Resorption in Monocyte Cultures Stimulated With M-CSF and RANKL

**Figure 4:**
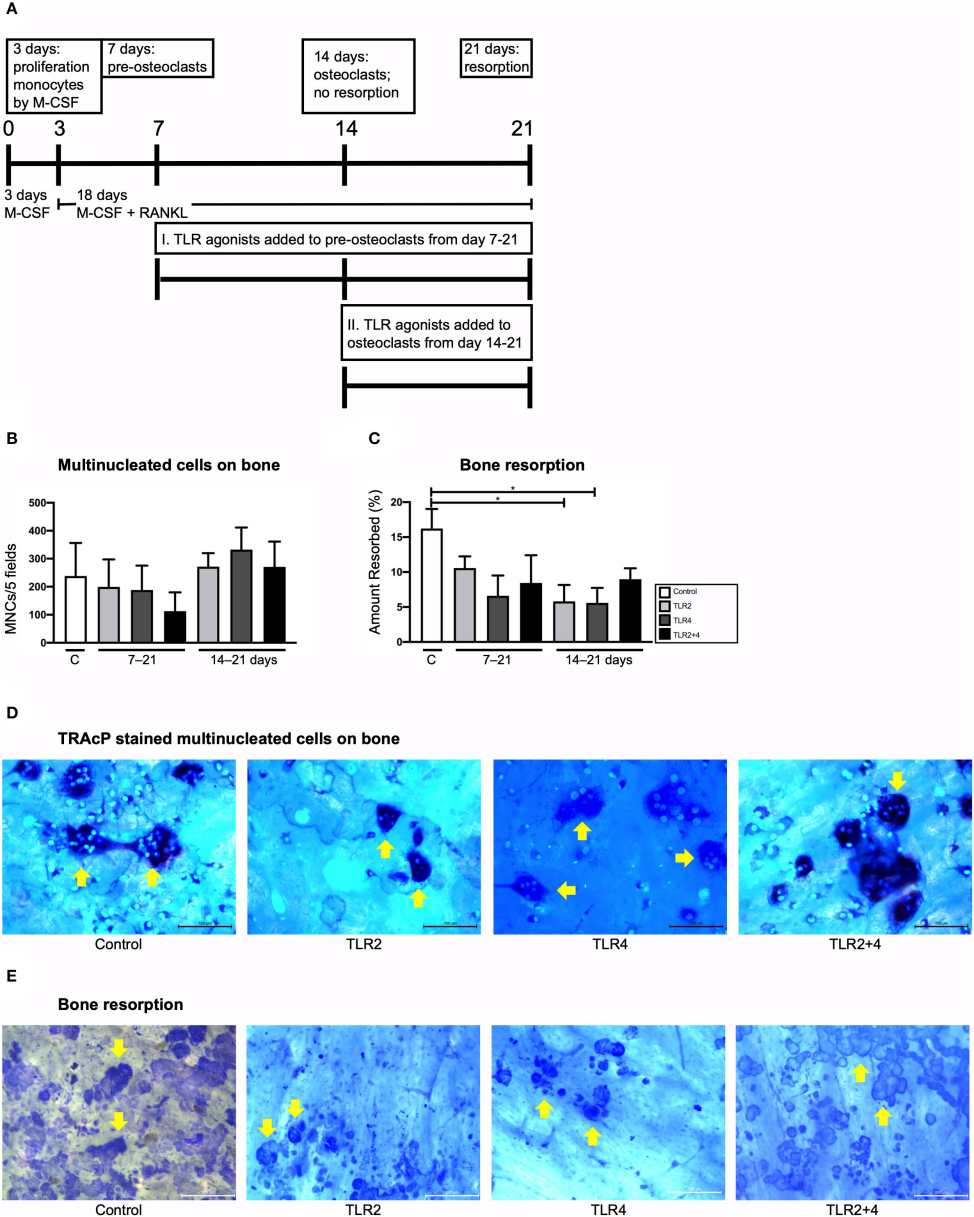
Effect of TLR agonists on the formation of multinucleated cells and on bone resorption in CD14+ monocyte cultures stimulated with M-CSF and RANKL. **(A)** Flow chart of the conducted experiment. Day 0–3: monocytes were cultured with M-CSF. Day 3–7: M-CSF + RANKL were added to the monocyte cultures. Day 7: Under the effect of M-CSF + RANL, monocytes have formed pre-osteoclasts. Control condition: no TLR agonists were added to this culture; Condition I.: TLR agonists were added to pre-osteoclasts from day 7 to 21 (chronic exposure on pre-osteoclasts); Condition II.: TLR agonists were added to osteoclasts from day 14 to 21 (chronic exposure on osteoclasts before resorption). Cultures were terminated after 21 days. **(B)** Osteoclasts were counted. Condition I and condition II are shown in the x-axes (7–21 and 14–21 days, respectively). Osteoclast formation did not differ between the conditions. **(C)** Bone resorption was measured. Addition of TLR agonists did not affect bone resorption when added at day 7 and had a slightly inhibitory effect on bone resorption when added from day 14–21 for both TLR2 and TLR4 agonist. **(D)** Shows light microscopy micrographs of the TRAcP stained cells, and the DAPI counterstained nuclei. Traces of bone resorption were regularly noticeable, for instance in the condition with TLR2 agonist. **(E)** Resorption pits were quantified after staining with Coomassie brilliant blue. Examples are shown from TLR agonist exposures from 14 to 21 days. Bars in micrographs represent 100 μm **(D)** and 200 μm **(E)**. Data from 1 out of 2 experiments are shown, similar results were obtained in both experiments. *n* = 4 per condition, in quadruplicates; average results ±SD are shown. Significant results for **(B,C)** are shown (black bars). *p < 0.05.

The above described osteoclastogenesis inhibitory effects by TLR2 and TLR4 agonists that were performed on cultures with naive monocytes, present in PBMCs that were stimulated with TLR agonists right after isolation. Although the GF-PBMC co-culture and the high-density PBMC cultures are good models to investigate the formation of multinucleated cells under the influence of GF or T-cells respectively, resorption by these multinucleated cells has never been observed (48, 51). In fact, the addition of M-CSF and RANKL is essential to achieve bone resorption (25). To further investigate TLR activation on osteoclast precursors of various stages of differentiation, TLR2 and TLR4 agonists were added to monocyte cultures that were stimulated with M-CSF and RANKL on day 7 (early stage osteoclast differentiation), day 14 (late stage, just before resorption takes place), or not at all (control condition) (Figure 4A). Cultures were terminated after 21 days. The cells were stained for TRAcP activity and DAPI and cells with 3 or more nuclei were counted (depicted with yellow arrows in Figure 4D). The addition of TLRs on day 7 and day 14 was not associated with a change in the number of multinucleated cells (Figures 4B,D which depicts condition II). For the cultures that were cultured on bovine bone slices (Figure 4E), bone resorption was quantified (Figure 4C). Bone slices were stained with Coomassie Brilliant blue and resorption pits were identified and their surface was measured using Image-Pro Plus software (Media Cybernetics, Silver Spring, MD). Bone resorption was hardly affected when TLR agonists were added, both when added at 7 or at 14 days. The only conditions that differed statistically with the control were TLR2 and TLR4, both when added on day 14. These experiments show that chronic exposure to TLR2 or TLR4 agonists did not affect osteoclast formation from osteoclast precursors or already formed osteoclasts. Furthermore, no increase in bone resorption was observed, rather slightly decreased bone resorption compared to conditions without TLR activation (Figure 4C).

### GFs Express TLR2 and TLR4 but at a Lower Level Compared to Osteoclasts

**Figure 5:**
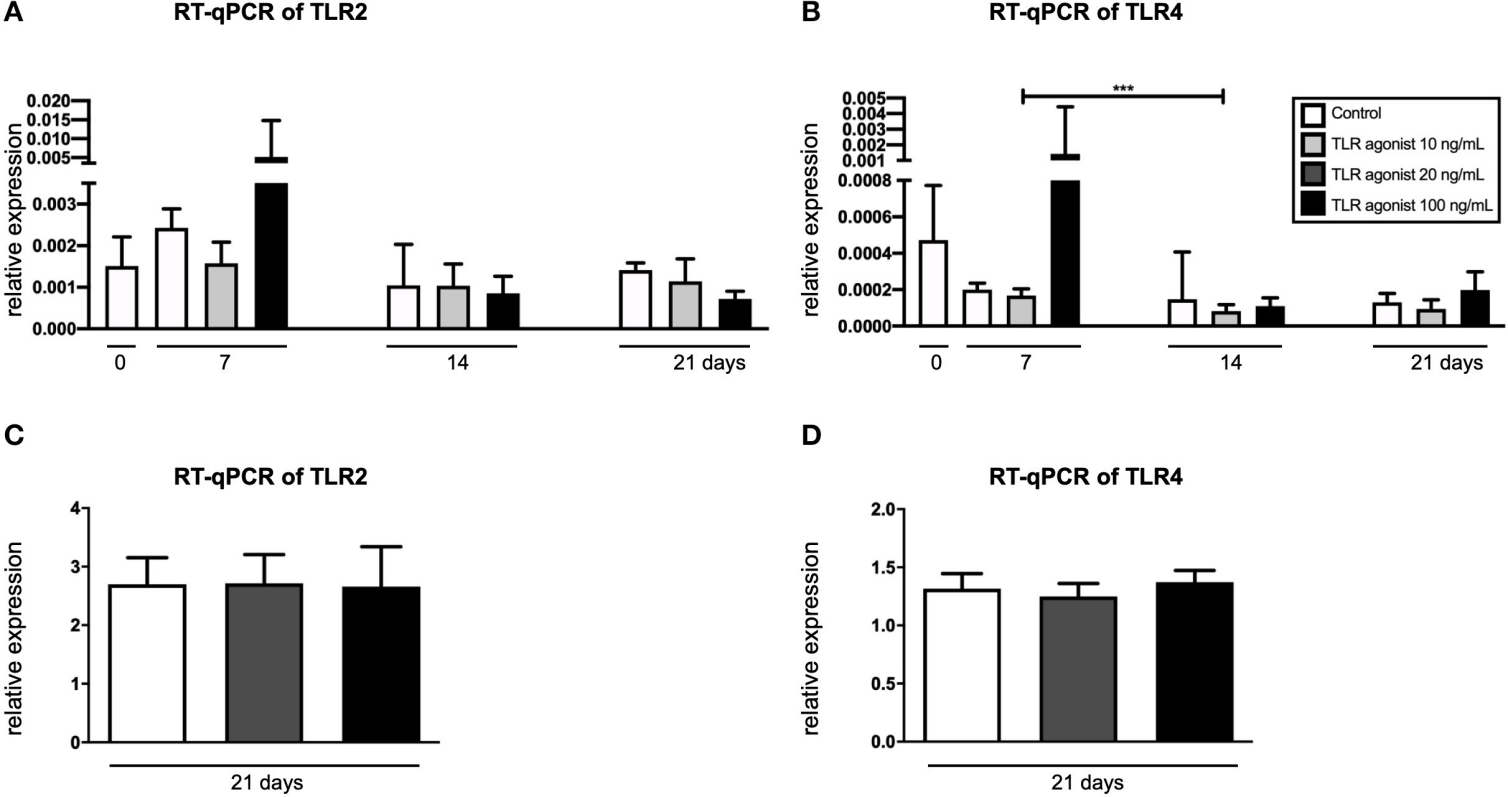
GFs and monocytes express TLR2 and TLR4. **(A)** Expression of TLR2 and **(B)** TLR4 in GFs cultures (*t* = 0) and co-cultures of GFs and PBMCs (*t* = 7, 14, and 21). **(C)** Expression of TLR2 and **(D)** TLR4 in cultures of CD14+ cells, cultured with M-CSF and RANKL. LPS-PG (Invivogen, San Diego, CA, USA) was used as TLR2 and TLR4 agonist in concentrations of 10 ng/mL or 20 ng/mL and 100 ng/mL. *n* = 6 for the co-cultures and *n* = 4 for the CD14+ cells' culture; average results ±SD are shown. Significant results are shown (black bars). ***p < 0.001.

In order to confirm the expression of TLR2 and TLR4 by the cells of interest, RT-qPCR was performed (Figure 5). Co-cultures and CD14+ cultures stimulated with M-CSF and RANKL were cultured with a Pg-LPS that targets both TLR2 and TLR4, depending on the concentration. TLR2 is activated from 10 ng/mL, TLR4 from 100 ng/mL. Both these concentrations in M-CSF and RANKL stimulated monocyte cultures were used. The expression of TLR2 and TLR4 in mono-cultures of GFs (*t* = 0) and in co-cutures of GF-PBMCs was measured at 3 different time points (*t* = 7, 14, and 21 days), after triggering with Pg-LPS that targets both TLRs at concertations of 10 ng/mL or 20 ng/mL and 100 ng/mL (Figure 5A). No differences were found between the conditions, indicating that TLR2 and TLR4 are expressed constantly over time, independent of the LPS concentration. The only significant difference was a reduction of the expression of TLR4 from day 7 to day 14, at the concentration of 10 ng/mL. The expression of TLR2 and TLR4 was measured also in CD14+ cultures that were stimulated with MCS-F and RANKL, at 21 days, the timepoint when all cells are in the osteoclast lineage, either as TRAcP mononucleated or as multinucleated cells (Figures 5C,D). Osteoclasts expressed both TLR2 and TLR4, at a much higher level than GF or GF-PBMC co-cultures.

### TLR2 and TRL4 Agonists Do Not Affect the Osteogenic Capacity of the Gingival Fibroblasts

**Figure 6:**
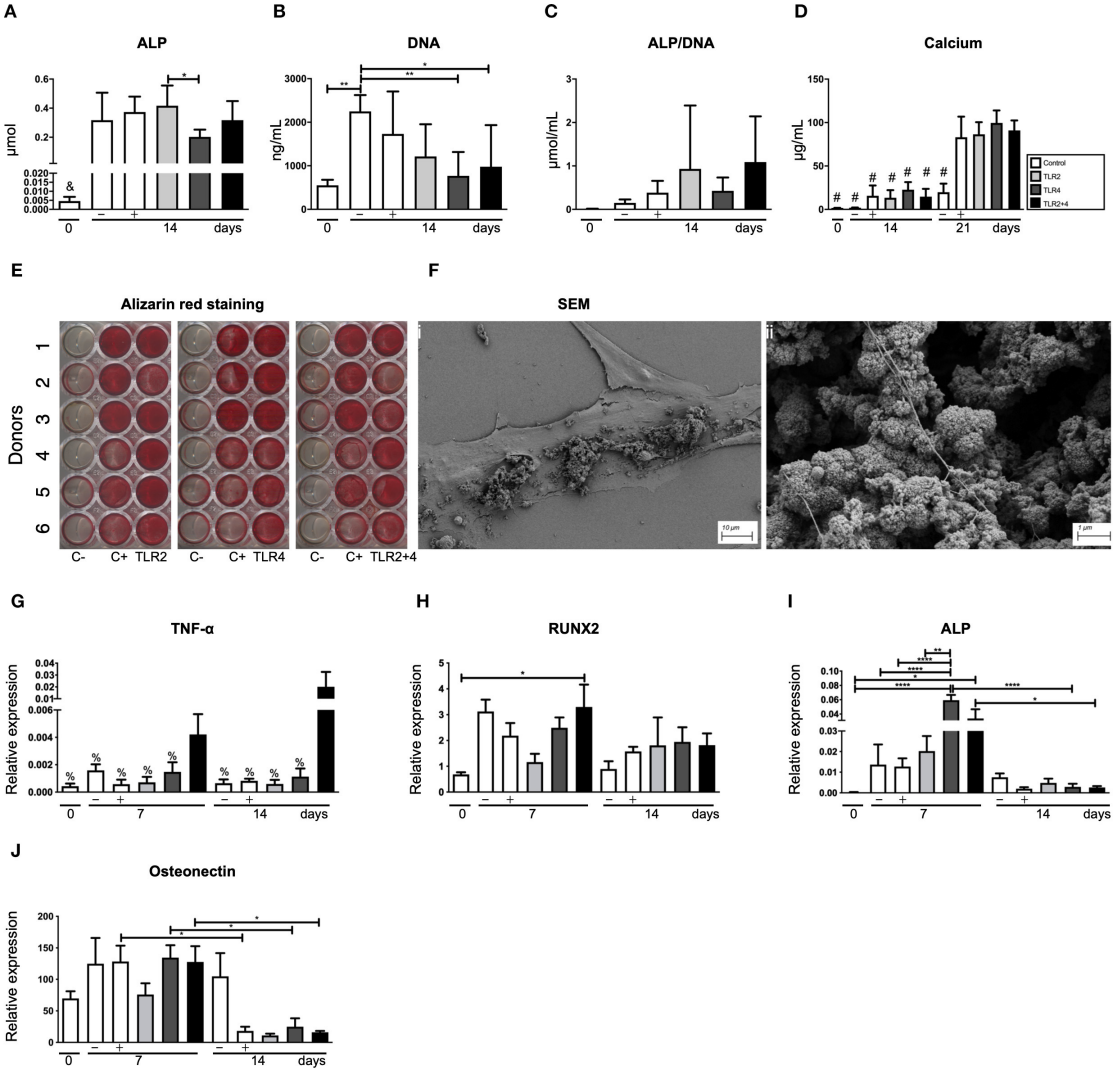
TLR agonists do not affect the osteogenic capacity of gingival fibroblasts. **(A)** Alkaline phosphatase (ALP), **(B)** DNA, **(C)** alkaline phosphatase corrected per number of cells (ALP/DNA), and **(D)** calcium deposition were quantified. **(B)** Compared to control cultures, TLR4 agonist (alone or with TLR2) affected the proliferation of cells. Overall, no significant differences were observed between the control conditions and the TLR agonists for **(A)** ALP, **(C)** Calcium deposition, and **(E)** Alizarin red staining. Alizarin red staining was done using control conditions without (C–) or with (C+) mineralization medium. The staining showed heterogeneity between the 6 donors. **(F**i**)** Shows the osteogenic matrix under scan electron microscopy and **(F**ii**)**presents structures that resemble mineral nodules. **(G–J)** Present the results of the quantitative PCR. **(G)** TNF-α is positively mediated by the combination of TLR2+4 at early and late time points (day 7 and 14). **(H)** RUNX2 is only increased at day 7 by the combination of TLR2+4, in comparison with the baseline (day 0). **(I)** Expression of the ALP gene is elevated in all conditions at day 7 in comparison with the baseline (day 0). TLR4 is also elevated compared to the negative and positive controls at day 7 and compared to the TLR4 at day 14. TLR2+4 reduced significantly from day to day 14. **(J)** Osteonectin gene expression reduced significantly from day 7 to day 14 for the control, TLR4 and TLR2+4. *n* = 6 per condition in duplicates, average results ±SD are shown. Significant results for **(A–D)** and **(G–H)** are shown (black bars). *p < 0.05, **p < 0.01, ***p < 0.001, ****p < 0.0001 in comparison to all the other conditions, ^#^*p* < 0.0001 in comparison to the c (+), TLR2, TLR4, and TLR2+4 at day 21, ^%^*p* < 0.001 in comparison to TLR2+4 at day 14.

To establish the effect of TLR2 and TLR4 agonists on osteogenesis, different osteogenic assays were conducted. Alkaline phosphatase and DNA content were measured at baseline (day 0) and day 14, with and without osteogenic medium (Figure 6A). There were no significant differences between the control and the other conditions on day 14. However, the addition of especially TLR4 agonists seemed to reduce the number of cells on day 14 (Figure 6B). The calculated ALP/DNA, or alkaline phosphatase corrected per number of cells, was not significantly different, with a lot of variation between the conditions (Figure 6C). Deposited calcium was measured at three different time points (*t* = 0, 14, and 21 days, Figure 6D). On day 14 and 21, calcium was only measured in conditions cultured in osteogenic medium and the concentration of calcium increased between day 14 and 21. However, the addition of TLR agonists did not influence calcium deposition (Figure 6D). Alizarin red staining confirmed these findings; no effect of TLR activation was observed (Figure 6E). Mineralization was confirmed with scanning electron microscopy (SEM, Figure 6F). Mineralization was present on top of cells (Figure 6Fi), as nodular structures, sometimes containing fibrillar structures, reminiscent of bone matrix proteins such as collagen I (Figure 6Fii).

Unlike the osteoclastogenesis experiment with co-cultures, TNF-α protein was undetectable in the supernatants of osteogenic cultures stimulated with TLR agonists (Figure 6G). However, at the mRNA level, low expression of TNF-α was detected, only significantly higher expression was found when TLR2 and TL4 agonists were added together at day 14 (Figure 6G). Early osteogenic marker RUNX2 was upregulated compared to t = 0 only in cultures with both TLR2 and TLR4, but no differences were found between conditions per time point, or between 7 and 14 days (Figure 6H). Intermediate marker ALP was upregulated at 7 days, especially in conditions where TLR4 agonist was added. As expected for ALP, the expression was lower at 14 days. No significant differences were observed between the conditions at 14 days (Figure 6I). Remarkably, late osteogenic marker osteonectin was significantly higher expressed at 7 days compared to 14 days (Figure 6J). Between conditions per time point, no significant differences were observed. Overall, influences of TLR agonists were limited in all gene expression analyses (Figures 6G–J).

## Discussion

Chronic diseases associated with bacterial pressure, such as periodontitis, are likely to experience phases of chronic exposure to bacterial products such as TLR activators. The effects of chronic exposure of cell cultures to TLR activators have been grossly neglected. In the present article, we describe the effects on osteoclast formation and activity on the one hand and on the osteogenic aspects on the other hand in cultures of GF that were chronically exposed to agonists of TLR2, TLR4, and their combination. TLR2 and TLR4 are the predominant TLRs activated in periodontitis (12, 22, 52).

A key finding of our study is that osteoclast formation is inhibited by TLR agonists when freshly isolated PBMCs are used. This was observed both in the co-culture's studies using GF and in the so-called high-density cultures. One could interpret these results in terms of the necessities of the inflamed periodontium, where relatively naive migrating monocytes may be triggered to differentiate into macrophages to nullify the effect of the bacterial products. The TNF-α ELISA results are in support of such a view: co-cultures produced high levels at early time points, especially in the presence of TLR2 agonists.

Intriguingly and relevant for our understanding of the immune reactions that take place during an infection is our finding that continuous exposure to TLR activators does not alter osteoclast differentiation when first primed with M-CSF and RANKL, both when added at the pre-osteoclast stage of 7 days and when added at the osteoclast stage of 14 days. This could indicate that the TLR-related induction of bone resorption *in vivo* (28, 29, 38), is due to the activation of the inflammatory milieu rather than directly through the osteoclast. In other words, the TLR reaction could elicit local stimulators of osteoclast differentiation such as IL-1β (53, 54) or TNF-α (55). Though not assessed, our results make it unlikely that osteoclasts or osteoclast precursors will express autocrine levels if osteoclast activate themselves after long-term exposure to TLR activators.

To the best of our knowledge, this is the first study that investigates the effect of TLR agonists in osteoclastogenesis and osteogenesis in a model of chronic exposure. Additionally, it is the only study that evaluated both osteoclastogenesis and osteogenesis on human periodontal cells, and more specifically GF. There are a few studies (34, 42, 43) that have studied the osteogenic potential of human periodontal ligament cells (hPDL) exposed to TLR agonists, sometimes with conflicting results. In two independent studies (42, 43), hPDL cells were infected with *E. coli* LPS (TLR4 agonist) and it was found that the osteogenic capacity of the cells was reduced significantly. In another study (34), the effect of TLR ligands was investigated on hPDL cells. High doses of TLR1, TLR3, and TLR6 ligands inhibited the osteogenic potential of these cells. On the contrary, Albiero et al. (41) infected hPDL cells with *Porphyromonas gingivalis* LPS (TLR2 agonist) and found no additional effect on the osteogenic differentiation potential of these cells. However, in this study was not clearly stated if the TLR agonists were added in the culture media only once or also in every refreshment. The osteogenic gene analyses of the above studies were limited to 2 weeks of cultures. For Alizarin red staining, the cells were cultured for 21–28 days. In these experiments, we unequivocally showed no effect of TLR2, TLR4, or the combination of the two when taking into account parameters like alkaline phosphatase, calcium deposition or Alizarin red staining. TLR4 could have a slight influence on the level of ALP at mRNA levels, or, in combination with TLR2 on the mRNA expression of TNF-α. Our results are in line with the findings of Albiero et al. (41), as we also found that the addition of TLR2, TLR4, and the combination of those agonists do not affect the osteogenic potential of the GF. Of special interest: the qPCR data from the osteogenesis were only partly in line with what is commonly seen in osteogenic differentiation. RUNX-2 was highest at an early timepoint, demonstrating its early osteogenic differentiation character. ALP expression was surprisingly highest at day 7 and significantly so in all conditions compared to day 0. Under normal circumstances, ALP protein expression peaks at 14 days (14), apparently the enhanced protein expression is prepared 1 week earlier. Osteonectin expression is believed to be a late marker of osteogenic differentiation, not seen in our results where expression lowered at day 14. When comparing gene expression of all genes, it is remarkable that addition of mineralization medium seemed not to influence gene expression of osteogenic genes. Apparently, the inevitable increased cell density seen in the wells might in part control gene expression. TLR2 and TLR4 agonists did not change gene expression, but interestingly the combination of the two altered TNF-α expression, the only assay where a synergistic effect was seen in our study. The apparent different-from-expected expression patterns of ALP and osteonectin, could be due to the fact that gingival fibroblasts are less suited for osteogenic differentiation compared to periodontal ligament fibroblasts (56). Scanning electron microscopy confirmed that mineral nodules were formed by the GF. A previous study (57) showed that it is possible to isolate and culture mesenchymal cells from human GF, which showed osteogenic differentiation capacity.

With respect to osteoclastogenesis, our results reject the hypothesis of this paper. Based on *in vivo* studies in mice (38, 39, 58–60) which Pg-LPS have shown that TLR activation induces osteoclastogenesis and bone resorption, we hypothesized that the activation of the TLR ligands would lead to induction of osteoclasts formation and bone resorption. Our results present a mild inhibition of the formation of osteoclasts in the co-cultures and slight reduction of bone resorption. Ji et al. (30) studied osteoclastogenesis in monocytes cultures, primed with M-CSF, activated with TLRs or IFN-γ, and also in an *in vivo* murine model. They concluded that activation with TLR2 and TLR4 ligands results in inhibition of osteoclastogenesis via inhibition of RANK and CSF1R expression. In another study (61), they studied the formation of osteoclasts in murine bone marrow cells, activated with TLR2 and TLR4 ligands. These cells were primed with M-CSF + RANKL or only with M-CSF and the RANKL was added concomitantly with the TLR ligands (TLR2 and TRL4). They found that TLR ligands inhibited RANKL-mediated osteoclastogenesis when the ligand and RANKL were simultaneously added to the cultures. On the contrary, when the cells were primed with M-CSF and RANKL before the activation with the ligands, osteoclastogenesis was not arrested. In the recent review of Souza and Lerner (62), it is also supported that activation of TLR agonists in different stages affects differently the maturation of the osteoclasts. Concomitant addition of TLR agonists and RANKL at the stage of osteoclast progenitor cell leads to impaired osteoclastogenesis. On the contrary, osteoclast progenitor cells primed with RANKL and then activated with TLR agonists, in the absence of RANKL, differentiated into mature osteoclasts. Our results show an inhibition of the naive osteoclast precursor cells, whereas the already formed (pre-)osteoclasts were not affected, which is in line with these studies. As they state in the paper of Ji et al. (30), the inhibition of the osteoclastogenesis can be explained as a homeostatic reaction against the inflammatory effects. Another possible explanation of our findings, as stated earlier in the discussion, could be that the activation of the monocytes with the TLR ligands (and more specifically the TLR2) leads to the formation of more macrophages instead of osteoclasts. This scenario could also be related to highly increased concentrations of TNF-α that we found on day 3 in the conditions of TLR2 and TLR2+4.

Another interesting implementation of our study was the 21 days duration of our experiments. The previously mentioned osteoblasts cultures (34, 41–43) were executed as well in a 21 days' timeframe, as this time is needed for the formation of osteoblasts. Regarding the osteoclastogenesis, this is the first experiment that evaluated the effect of the TLRs in a timeframe of 21 days. Most of the studies that were performed studied the formation of osteoclasts in a timeframe of 2–5 days, typical for mouse osteoclasts (36–40), and in the studies of Kassem et al. (38) and Liu et al. (37), the bone resorption experiments had a duration of 6–7 days. This long exposure on the TLR agonists shows an effect on the expression of TNF-α. Accordingly, we found a higher production of TNF-α on the conditions of TLR2 and TLR2+4 on day 3, in comparison with the control and the TLR4 condition. This finding was reversed on day 7, where TLR2 and TLR2+4 were significantly lower than the control. On day 21, the only remaining difference we observed, was between TLR2 and TLR4 conditions, with the former one being higher. Apparently, the co-culture cell system normalizes over time: at 3 days all cultures containing TLR2 agonists responded with increased TNF-α secretion, followed by the reverse on day 7 and no differences between the conditions at day 21.

In this study, we investigated the chronic effect of the TLR2 and TLR4 agonists on osteoclastogenesis and osteogenesis, measuring several parameters. When observing an effect, it was often TLR2 agonist-mediated. The ability of naive osteoclast precursor cells to form osteoclasts, for instance, was consistently inhibited by TLR2 agonists. Bone resorption also appeared to be reduced by the addition of TLR2 agonists when added at a late time point (day 14). TLR2 had also an evident effect on the production of TNF-α, playing an enchasing role on day 3, which was reversed at the later time points (days 14 and 21). At gene expression level, expression of NFATc1, a key transcription regulator of osteoclast differentiation (50), was downregulated on day 7 by TLR2 agonist. The only effect of TLR4 on osteoclastogenesis was a slight reduction of the bone resorption when it was added at a late time point (day 14). Furthermore, it affected ALP gene expression, with an enhanced expression at day 7. The combination of TLR2+4 agonists only enhanced TNF-α gene expression during osteogenesis, both at days 7 and 14.

**Figure 7:**
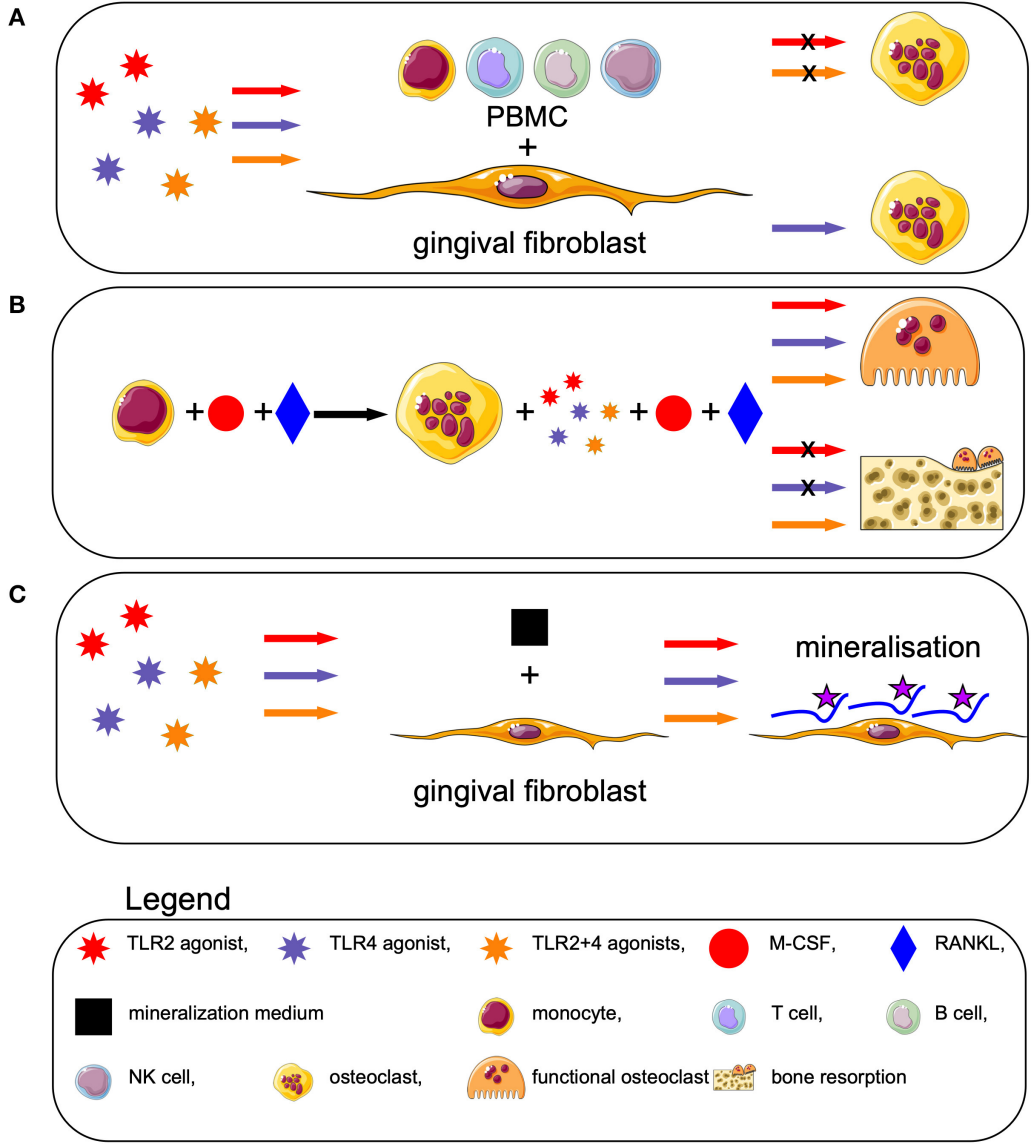
TLR activation affects osteoclastogenesis differently, depending on the differentiation stage of the blood cells, but does not affect osteogenesis. **(A)** Activation of human peripheral blood mononuclear cells (PBMCs) with TLR2 and TLR2+4 agonists, in presence of GF, decreases the formation of osteoclasts. **(B)** Priming of monocytes with M-CSF + RANKL leads to formation of pre-osteoclast. Triggering of these cells with TLRs in an early stage (from day 7 to 21), concomitant with M-CSF and RANKL, induces formation of bone resorbing osteoclasts. When the TLRs are added on a later stage (from day 14 to 21), the bone resorption capacity of the osteoclasts is reduced. **(C)** Triggering of GF with TLRs, in presence of mineralization medium, precipitates osteogenesis.

In our previous study (24), we demonstrated that GF and monocytes can mediate the diversity of the cellular populations at the site of inflammation, by reducing the number of B-, T- and NK-cells. In this aforementioned study, we also showed that TLR2 activation is an important player in T cell proliferation in the presence of monocytes (20). In the current study, we showed that activation with TLR agonists of naive or M-CSF and RANKL primed human osteoclast precursors has differential effects. This could indicate that fresh monocytes that encounter bacterial products such as TLR2 or TLR4 agonists, may differentiate into macrophages that eradiate these inflammation activators (Figure 7). Osteoclasts could be the cells that are NOT activated by TLR directly, but rather indirectly, through the microenvironment's expression of inflammatory cytokines that are known activators of osteoclasts.

## Data Availability Statement

The raw data supporting the conclusions of this article will be made available by the authors, without undue reservation.

## Ethics Statement

The studies involving human participants were reviewed and approved by VUmc. The patients/participants provided their written informed consent to participate in this study.

## Author Contributions

GK and TV designed the experiments. GK was involved in collecting most of the data, some of them were retrieved under the supervision of IJ, TS, JH, HV, and CM. GK and TV performed the TLR titration experiments together, results were analyzed by GK. TV pipetted the experiment with co-cultures and osteogenesis. ES and TV designed and performed the experiments with monocytes stimulated with M-CSF and RANKL and TLR agonists (Figure 4), analyzed by ES. KŁ-Ć and TV designed and performed the experiment of the expression of TLRs (Figure 5). Wisdom teeth, essential for all experiments involving GF, were collected by TF. GK initiated writing, first drafts were corrected by TV, and all authors have commented on the final version and agree with the present version.

## Conflict of Interest

The authors declare that the research was conducted in the absence of any commercial or financial relationships that could be construed as a potential conflict of interest.
